# Artificial Intelligence and Machine Learning in Audiology and Hearing Disorders: A Scoping Review with Bibliometric and Thematic Mapping (1995–2025)

**DOI:** 10.3390/audiolres16020029

**Published:** 2026-02-24

**Authors:** Ceren Aksoy Koçak

**Affiliations:** Department of Otorhinolaryngology–Head and Neck Surgery, Konya City Hospital, University of Health Sciences, Konya 42090, Türkiye; ceren.aksoykocak@saglik.gov.tr

**Keywords:** artificial intelligence, machine learning, audiology, hearing disorders, scoping review, bibliometric analysis

## Abstract

**Background and Objectives:** Artificial intelligence (AI) and machine learning (ML) are increasingly integrated into audiology, supporting diagnosis, screening, rehabilitation, and digital health. Despite rapid growth, the literature remains methodologically and clinically heterogeneous, limiting a consolidated view of research trajectories and translational readiness. This scoping review examined the evolution of AI and ML applications in audiology and hearing disorders, focusing on thematic development, research productivity, collaboration patterns, and clinical orientation. **Methods:** A scoping review was conducted using the Web of Science Core Collection (Science Citation Index Expanded). Original and review articles published between 1995 and 2025 were included. Bibliometric and thematic mapping were applied to analyze publication trends, citation patterns, keyword evolution, and collaboration networks. A structured translational categorization assessed clinical domains and validation maturity. Findings reflect the Web of Science-indexed segment of the literature. **Results:** A total of 127 publications were analyzed. Research output increased markedly after 2020, with an estimated doubling time of approximately 2.1 years. China, the United States, and South Korea contributed the highest publication volumes, although citation impact did not consistently parallel productivity. Thematic analyses revealed a shift toward AI-driven methodological frameworks, particularly in machine learning, deep learning, and cochlear implant-related applications. Most studies remain at proof-of-concept or internally validated stages, with limited external validation. Emerging areas include tele-audiology and personalized hearing aid optimization. **Conclusions:** AI and ML research in audiology is increasingly application-oriented; however, broader external validation and prospective implementation are required to support routine clinical integration.

## 1. Introduction

Artificial intelligence (AI) and machine learning (ML) have increasingly reshaped contemporary medical research by enabling data-driven diagnostic, prognostic, and therapeutic decision-making. Early clinical implementation was particularly evident in imaging-based specialties such as ophthalmology and dermatology, where deep learning systems achieved specialist-level diagnostic performance [1,2]. With advances in computational infrastructure and algorithmic architectures, AI/ML methodologies have expanded across diverse medical domains, influencing both research paradigms and clinical workflows.

In audiology, AI and ML applications span a broad range of clinical and research contexts. Machine learning-based classification models have been developed for automated diagnosis of external and middle-ear conditions [3], auditory signal processing, and automated hearing assessment workflows using spectrogram-based representations [4,5]. Predictive modeling has been explored in occupational noise-induced hearing loss [6], while AI-driven systems have contributed to cochlear implant optimization and electrophysiological threshold estimation [7]. Tele-audiology frameworks and digital health platforms further support remote screening, diagnosis, and rehabilitation [8]. The multidimensional nature of auditory disorders—acoustic, electrophysiological, perceptual, and behavioral—renders audiology particularly suitable for integrative data-driven modeling.

The global burden of hearing loss underscores the relevance of these technological developments. More than 1.5 billion individuals worldwide experience some degree of hearing loss, with projections reaching 2.5 billion by 2050 [9]. This growing prevalence highlights the need for scalable and cost-effective diagnostic and rehabilitative strategies, increasing interest in AI-supported screening and automated classification systems [10,11,12].

Despite rapid expansion, AI/ML research in audiology remains methodologically heterogeneous and dispersed across clinical and technical subdomains. Existing reviews have often focused on specific applications, such as imaging tasks or cochlear implant-related modeling [13], while recent scoping reviews have summarized contemporary applications and translational challenges [14]. However, a comprehensive bibliometric and thematic mapping analysis that quantitatively evaluates publication growth, collaboration structures, citation dynamics, and translational maturity within audiology-specific AI/ML research remains lacking.

The present study, therefore, conducts a scoping review of AI and ML applications in audiology and hearing disorders from 1995 to 2025. By integrating bibliometric and thematic mapping approaches, this review aims to: (i) characterize publication growth and citation patterns; (ii) identify dominant and emerging research themes; (iii) examine geographic and institutional collaboration networks; and (iv) assess the translational orientation and validation maturity of published applications.

## 2. Materials and Methods

### 2.1. Study Design

This study was designed as a scoping review of the literature addressing artificial intelligence (AI) and machine learning (ML) applications in audiology and hearing disorders. Bibliometric and network analyses were used as complementary analytical tools to describe research trends, thematic evolution, and collaboration patterns, in line with the objectives of a scoping review framework.

The review was conducted in accordance with the Preferred Reporting Items for Systematic Reviews and Meta-Analyses extension for Scoping Reviews (PRISMA-ScR) guidelines. The study selection process is summarized in the PRISMA-ScR flow diagram (Supplementary Figure S1).

### 2.2. Data Source and Search Strategy

The literature search was performed in the Web of Science (WoS) Core Collection, Science Citation Index Expanded (SCI-E) database on 5 August 2025. WoS was selected due to its standardized indexing structure, comprehensive citation data, and suitability for citation-based bibliometric and network analysis, which require consistent metadata and reference linkage.

The following search strategy was applied in the Topic Search (TS) field:

(“Artificial Intelligence” OR “Machine Learning” OR “Deep Learning”) AND (“hearing loss” OR audiology OR “hearing aid” OR “cochlear implant” OR “tele-audiology”) AND (diagnosis OR detection OR screening OR rehabilitation)

To ensure search specificity, truncation symbols were not used. Only publications written in English, indexed in SCI-E, and categorized as original articles or review articles were included. No restrictions were applied regarding publication year. The earliest eligible record was published in 1995.

The complete search strategy and keyword combinations are provided in Supplementary Table S1.

Because bibliometric analyses require standardized citation metadata and reference linkage, only WoS-indexed records were included; therefore, the findings represent the WoS-indexed segment of the literature rather than the entirety of AI/ML research in audiology.

### 2.3. Study Selection and Data Cleaning

The initial search yielded 162 records. Titles and abstracts were screened to exclude irrelevant studies, including publications focused primarily on linguistics, general hearing science, or non-audiology-related neuroscience without explicit AI/ML methodology.

Misclassified records (e.g., review articles indexed as original articles) were manually corrected. After duplicate removal and eligibility assessment, 127 publications (111 original articles and 16 review articles) were included in the final analysis. The study selection process was documented using a PRISMA-ScR flow diagram (Supplementary Figure S1).

### 2.4. Data Analysis and Bibliometric Methods

Bibliometric and network analyses were conducted using VOSviewer (version 1.6.19) and the Bibliometrix/Biblioshiny package (R version 4.3.1). Analytical procedures followed established bibliometric guidelines [15].

Analyses included annual publication trends, country and institutional productivity, author and journal contributions, citation impact, keyword co-occurrence, thematic mapping, and co-citation network analysis. Keyword co-occurrence analysis was conducted to identify conceptual structures and thematic clusters within the field, based on established co-word analysis principles [16]. Thematic mapping and strategic diagram analysis were performed using sciencemapping techniques grounded in centrality and density metrics, following recognized methodological frameworks for bibliometric visualization and theme evolution analysis [17].

In addition to bibliometric indicators, records were descriptively categorized according to clinical application domain and reported validation stage to summarize translational characteristics of the included studies.

Descriptive indicators were evaluated separately for original articles and review articles. Network-based analyses—including collaboration networks and thematic evolution—were performed on the combined dataset. The full-counting method was applied, assigning equal credit to each contributing author, institution, and country.

Publication growth trends were modeled using exponential regression analysis. Doubling time (DT) was calculated using the equation DT = ln(2)/β, where β represents the regression coefficient. Model goodness-of-fit was assessed using R^2^ values.

## 3. Results

### 3.1. Annual Distribution of Publications

**Figure 1:**
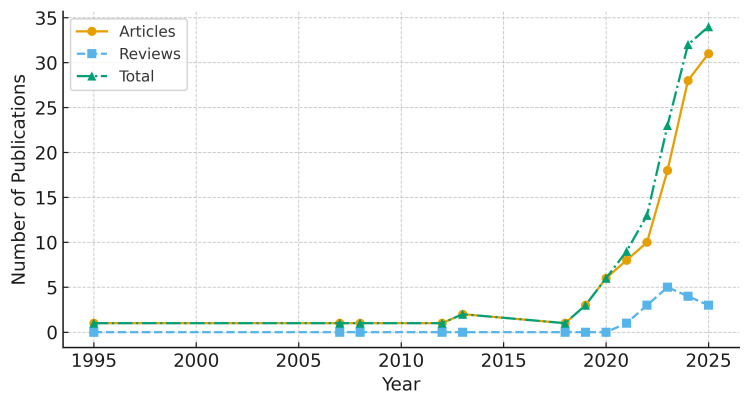
Annual number of publications on artificial intelligence (AI) and machine learning (ML) in audiology and hearing disorders (1995–2025). The number of publications increased exponentially, doubling approximately every 2.1 years, with a sharp rise after 2020.

A total of 127 publications were included in the analysis, comprising 111 original research articles and 16 review articles. The earliest eligible publication appeared in 1995. Publication output remained limited until 2019, followed by a marked increase after 2020. Peaks were observed in 2023 (n = 23), 2024 (n = 32), and 2025 (n = 34) (Figure 1).

Review articles increased notably after 2021. Exponential regression analysis demonstrated that annual publication output doubled approximately every 2.1 years. Detailed yearly publication counts are provided in Supplementary Table S2.

### 3.2. Country Productivity

China contributed the largest number of publications (n = 34), followed by the United States (n = 20) and South Korea (n = 14). Germany, Turkey, and the United Kingdom also showed notable research output.

The ten most productive countries are summarized in Table 1. Separate distributions for original articles and review articles are presented in Supplementary Tables S3 and S4 and Supplementary Figure S2.

### 3.3. Institutional Productivity

At the institutional level, Carl von Ossietzky University of Oldenburg (Germany) was the most productive institution, contributing four publications. Several institutions from South Korea, China, and the United States contributed three publications each, while multiple other institutions contributed two publications.

A complete list of productive institutions is provided in Supplementary Table S5.

### 3.4. Citation Impact of Countries and Institutions

At the country level, the United States, China, the United Kingdom, and South Korea ranked among the most productive contributors. However, citation impact analysis revealed a different pattern. Chile ranked first in citation impact, largely driven by a single highly cited publication (n = 1; 57 citations; h-index = 1).

In contrast, the United Kingdom (n = 7; h-index = 4) and South Korea (n = 6; h-index = 2) demonstrated more consistent citation performance (Supplementary Table S6).

At the institutional level, Radboud University Nijmegen achieved the highest citation count (n = 4; 176 citations; h-index = 4), followed by the University of Erlangen–Nuremberg and the University of Iowa (Supplementary Table S7). Industrial and academic entities such as Cochlear and Universidad de Antioquia also exerted substantial influence through single high-impact publications. Author-level citation metrics are detailed in Supplementary Table S8.

### 3.5. Most Influential Journals and Bradford’s Law Analysis

Original research articles were most frequently published in Scientific Reports (n = 8), Hearing Research (n = 6), and Frontiers in Neuroscience (n = 6). Other prominent outlets included Otolaryngology–Head and Neck Surgery (n = 5), Ear and Hearing (n = 4), Applied Sciences–Basel (n = 4), and Diagnostics (n = 3). Among review articles, the Journal of Clinical Medicine (n = 3) was the most represented.

Bradford’s law analysis identified three productivity zones: Zone 1 (9 journals, 43 publications), Zone 2 (29 journals, 42 publications), and Zone 3 (42 journals, 42 publications), as summarized in Table 2.

### 3.6. Keyword Analysis

Across original research articles, 392 unique Author Keywords were identified, while review articles contributed 79 unique keywords. In original articles, the most frequent Author Keywords were machine learning (n = 31), deep learning (n = 22), and hearing loss (n = 17). In review articles, artificial intelligence (n = 9) and machine learning (n = 8) predominated.

Keywords Plus analysis revealed substantial overlap between study types, with hearing loss, classification, and diagnosis appearing most frequently. Original articles emphasized clinical terms (e.g., adults, noise, recognition), whereas review articles highlighted methodological concepts such as neural networks, management, and prediction. The most frequent terms are summarized in Table 3.

### 3.7. Temporal Trends in Keywords

A temporal analysis of the ten most frequently used keywords is shown in Supplementary Figure S3. Prior to 2018, clinically oriented terms such as hearing loss and cochlear implant predominated. From 2019 onward, methodological terms—particularly machine learning and deep learning—increased sharply. After 2021, machine learning emerged as the most dominant keyword.

### 3.8. Collaboration Networks

Co-authorship network analysis identified Liu, Xiao, Schilling, Achim, and Shew (Matthew) as central authors linking multiple collaborative clusters. At the institutional level, the University of Cincinnati, the University of Iowa, Soonchunhyang University, and Radboud University Nijmegen functioned as major hubs.

International collaboration was strongest between the United States and China, with high network centrality also observed for the United Kingdom and Germany. Network visualizations are presented in Supplementary Figures S4–S6.

### 3.9. Most Cited Publications and References

The most cited publication was authored by Arias-Vergara et al. (65 citations), followed by Botros et al. (Artificial Intelligence in Medicine, 63 citations) and Heutink et al. (Computer Methods and Programs in Biomedicine, 55 citations). Other highly cited studies focused on ML-based diagnosis of ear disease, deep learning detection of otitis media, and prediction of noise-induced hearing loss.

The ten most cited publications are summarized in Supplementary Table S9.

The fifteen most frequently cited references are presented in Supplementary Table S10 and illustrated in Supplementary Figure S7. Prominent methodological milestones included ResNet, DenseNet, Random Forests, LSTM, and scikit-learn, alongside domain-specific audiological studies. This distribution highlights the dual reliance on methodological innovation and clinical evidence.

### 3.10. Co-Citation and Thematic Analyses

Article-level co-citation analysis identified three major thematic clusters: AI-based audiological assessments, cochlear implants, and methodological or bibliometric studies (Supplementary Table S11).

**Figure 2:**
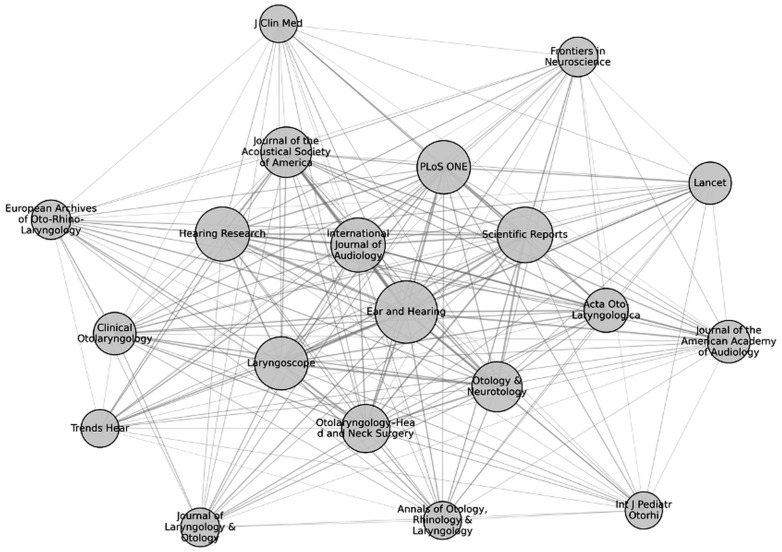
Journal-level co-citation network of AI/ML research in audiology. The network reveals three main clusters representing methodological, clinical, and mapping-related foci. *Ear and Hearing, Scientific Reports*, and *Hearing Research* occupy central positions, indicating strong interdisciplinary influence.

At the journal level, the strongest co-citation links involved Ear and Hearing, Scientific Reports, Hearing Research, International Journal of Audiology, and PLOS ONE (Figure 2; Supplementary Table S12). Clinically oriented journals also occupied central positions within the network.

**Figure 3:**
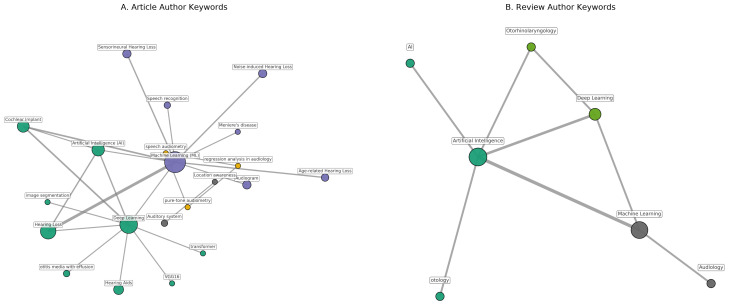
Thematic clusters based on Author Keywords. (**A**) Research articles show four clusters: (i) AI/ML methods; (ii) hearing loss subtypes; (iii) audiological assessment; and (iv) diagnostic/predictive approaches. (**B**) Review articles form three clusters focusing on AI/ML techniques, clinical applications, and health technologies such as tele-audiology.

Thematic analysis based on Author Keywords revealed four clusters in the original research articles:

(i) AI/ML methodologies;
(ii) hearing loss subtypes;
(iii) audiological assessment techniques;
(iv) diagnostic and predictive approaches (Figure 3A; Supplementary Table S14).

Review articles clustered into three thematic groups encompassing AI/ML methods, clinical applications, and health technologies (Figure 3B; Supplementary Table S16).

**Figure 4:**
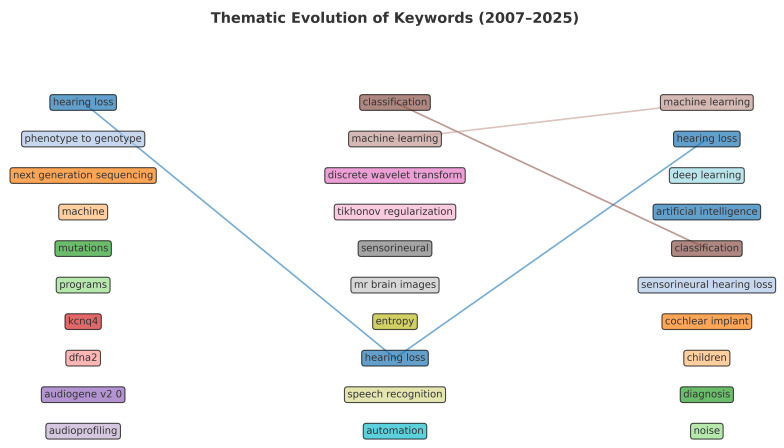
Thematic evolution of AI/ML research in audiology (2007–2025). Thematic development shifted from clinically oriented concepts (hearing loss, cochlear implant) in early phases to methodological topics (deep learning, neural networks, tele-audiology) after 2019, reflecting the field’s maturation and translational orientation.

Thematic evolution analysis demonstrated a shift from clinically focused topics during 2007–2019 toward methodological dominance after 2019 (Figure 4).

**Figure 5:**
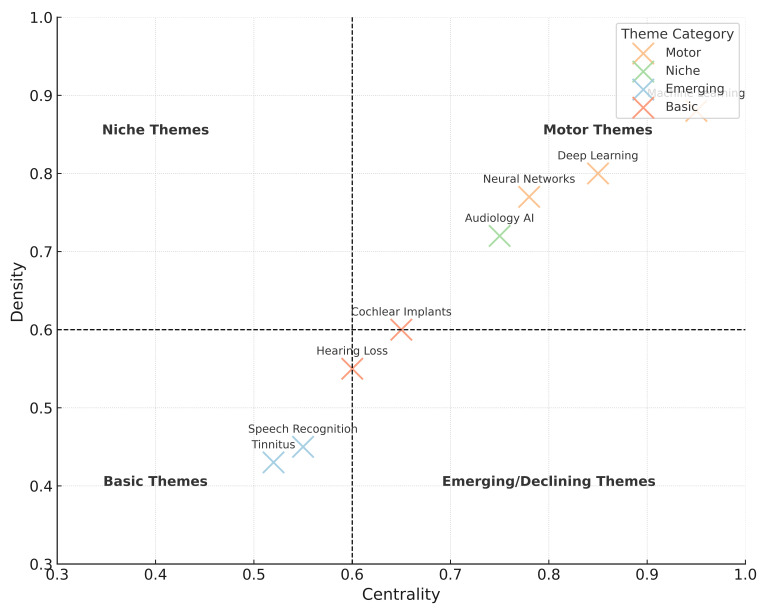
Thematic map of major research domains in audiology-related AI/ML publications (2020–2025). Motor themes include deep learning and cochlear implant; artificial intelligence and audiology appear as basic and expanding themes; tinnitus and high-frequency audiometry emerge as niche themes; and hearing aid fitting and screening tools represent developing themes.

Thematic mapping showed *machine learning* and *hearing loss* as motor themes in earlier periods, while *deep learning* and *cochlear implant* emerged as motor themes in 2020–2025. Emerging and niche themes included high-frequency audiometry, tinnitus, hearing aid fitting, and screening tools (Figure 5).

### 3.11. Funding Agencies and Open Access Characteristics

The most frequently acknowledged funding sources included the National Key Research Program, the BK21 FOUR initiative, and the Soonchunhyang University Research Fund, each supporting multiple publications. Additional funding originated from national ministries and institutional grants in South Korea, China, and the United States (Supplementary Table S18).

Regarding accessibility, 65.4% of publications were available through some form of open access. Gold open access accounted for the largest share (41.7%), followed by Hybrid (12.6%) and Green (8.7%) access, while 2.4% were classified as Bronze. The remaining 34.6% were non-open access publications (Supplementary Table S19).

### 3.12. Clinical Application and Validation Characteristics

To provide additional insight into the translational orientation of AI/ML research in audiology, the included records (n = 127) were descriptively categorized according to (i) primary clinical application domain, (ii) predominant AI/ML approach, and (iii) reported validation stage, based on information available in titles, keywords, and abstracts. This structured categorization complements bibliometric and thematic analyses by summarizing the clinical focus and reported validation characteristics of published studies.

Across domains, the majority of publications were concentrated in hearing loss diagnosis and screening, followed by cochlear implant applications and noise-induced hearing loss prediction. Deep learning and machine learning approaches predominated across nearly all clinical domains. Most studies reported proof-of-concept designs or internal validation procedures, whereas externally validated or prospectively implemented clinical studies were comparatively limited. This distribution indicates a strong concentration of AI/ML research in diagnostic-oriented applications, while implementation-oriented studies remain limited.

Table 4 presents the distribution of AI/ML applications in audiology according to clinical domain, methodological approach, and reported validation stage.

Overall, this distribution highlights a strong concentration of AI/ML research in diagnostic and screening-oriented applications, while externally validated and clinically implemented studies remain comparatively limited within the current literature.

## 4. Discussion

In this scoping review, we mapped the evolution, thematic structure, and clinical orientation of artificial intelligence (AI) and machine learning (ML) research in audiology and hearing disorders over nearly three decades. Bibliometric mapping was used as a complementary analytical tool to contextualize research growth, collaboration patterns, and thematic development, rather than as an end in itself. Overall, the findings demonstrate a rapidly expanding and increasingly application-oriented research landscape, with clear implications for clinical translation.

Research output accelerated markedly after 2020, with publication volume doubling approximately every 2.1 years. This pattern parallels the broader expansion of AI/ML research across health sciences, as demonstrated in large-scale bibliometric analyses of artificial intelligence in medicine [18,19]. In parallel, domain-specific bibliometric investigations in otorhinolaryngology have reported increasing research activity in hearing-related and otologic AI applications [20]. Importantly, the observed expansion reflects not only methodological innovation but also increasing clinical engagement. Application-driven investigations have contributed to automated diagnosis of external and middle-ear pathologies [3], prediction of noise-induced hearing loss in occupational settings [6], and optimization of cochlear implant programming and electrophysiological threshold estimation [7]. Deep learning approaches have also been applied to imaging-based diagnosis of chronic otitis media using computed tomography [11]. Machine learning models have further enabled sensorineural hearing loss classification using signal-processing-based features [12], supporting more objective and reproducible diagnostic workflows. In parallel, digital and AI-supported tele-audiology models have emerged as scalable frameworks for remote assessment and follow-up [5,8], offering potential to reduce access disparities in underserved regions. Together, these patterns indicate a shift from exploratory algorithm development toward clinically motivated research embedded within real-world diagnostic and rehabilitative pathways. Nevertheless, despite increasing methodological sophistication, most studies remain at the proof-of-concept or internally validated stage, and prospective multi-center validation studies remain comparatively limited.

At the country level, China, the United States, and South Korea accounted for the largest share of publications, aligning with prior large-scale bibliometric analyses of artificial intelligence in medicine and healthcare, which have consistently reported China–US predominance in AI research output [18,19]. Domain-specific investigations in otorhinolaryngology similarly demonstrate increasing AI-related publication activity across hearing-related subfields [20]. While global AI research is often characterized by China–US dominance, our findings highlight South Korea’s balanced contribution in both output and citation impact. Notably, citation influence did not strictly parallel publication volume. This divergence between productivity and impact reflects established bibliometric observations indicating that publication quantity alone does not necessarily translate into sustained scientific influence [21]. Such patterns underscore the importance of jointly evaluating productivity and citation-based metrics when assessing research maturity, structural influence, and translational positioning in emerging interdisciplinary domains such as AI-driven audiology [22].

At the institutional level, several European and U.S. universities demonstrated high citation influence. Radboud University Nijmegen and the University of Erlangen–Nuremberg, in particular, achieved strong visibility through contributions that bridged methodological development and clinical application. Previous network-based bibliometric studies have demonstrated that institutions occupying central or interdisciplinary positions within collaboration and co-citation networks tend to achieve disproportionately higher scientific influence, not solely due to publication volume but through structural connectivity and cross-domain knowledge exchange [22,23]. Our findings support this model and emphasize the role of institutional networks in shaping clinically relevant AI research in audiology.

Collaboration patterns further reinforced the importance of multi-center and international partnerships. Author-level analyses indicated that productivity and citation visibility were largely driven by collaborative research networks. This observation aligns with established conceptual frameworks defining the structure and functions of research collaboration [24], as well as bibliometric evidence demonstrating a positive association between internationalization and scientific performance [25]. Beyond Asia, transatlantic collaborations emerged as particularly influential, suggesting that cross-regional partnerships may facilitate data sharing, methodological standardization, and more robust external validation processes. In clinically oriented fields such as audiology, such collaborative structures are especially critical for generating multi-center datasets, improving model generalizability, and accelerating the translation of AI-based tools into routine diagnostic and rehabilitative practice.

Journal-level analyses revealed that research activity was concentrated within a limited number of high-impact outlets, notably Scientific Reports, Hearing Research, and Frontiers in Neuroscience. This distribution aligns with Bradford’s law [26] and is consistent with clustering patterns described in prior bibliometric analyses of artificial intelligence in medicine [18,19] and otorhinolaryngology [20]. Importantly, specialty journals such as Ear and Hearing and Otolaryngology–Head and Neck Surgery maintained central positions within the co-citation network. Given that Ear and Hearing has historically functioned as a core platform shaping audiological research trajectories [27], its continued prominence in AI/ML publications suggests that data-driven methodologies are increasingly being integrated into established clinical research domains. This pattern is consistent with co-citation-based models, indicating that central journals contribute to structuring disciplinary knowledge and facilitating methodological diffusion within specialized fields [22].

Keyword and thematic analyses provided additional insight into the intellectual organization of AI/ML research in audiology. The frequent occurrence of terms such as machine learning, deep learning, and cochlear implant reflects the close interplay between computational methodology and clinically oriented audiological applications, consistent with prior domain-specific reviews describing the expanding role of AI in otologic research [13].

Thematic mapping further identified established motor themes—including hearing loss and machine learning—as well as emerging or developing themes such as tele-audiology and high-frequency audiometry. These findings suggest a progressive consolidation of core diagnostic applications alongside the gradual diversification of specialized subtopics within the field.

Temporal evolution analysis demonstrated a shift from predominantly clinically descriptive terminology in earlier periods toward increased emphasis on computational and methodological concepts in more recent years. This transition aligns with broader trend analyses reporting a methodological intensification of AI research within otorhinolaryngology and medical domains [20].

From a translational perspective, several application domains appear to be gaining increasing clinical relevance. These include automated diagnosis of hearing loss and otologic disorders [3,11,12], prediction of noise-induced impairment [6], and optimization of cochlear implant programming and electrophysiological threshold estimation [7]. Although many of these investigations remain at the proof-of-concept or internally validated stage, their growing frequency indicates an expanding interface between computational modeling and routine audiological assessment. However, the transition from algorithmic performance metrics to clinically meaningful outcome improvement remains insufficiently demonstrated. Prospective, multi-center validation studies incorporating heterogeneous patient populations are still limited, restricting generalizability and regulatory readiness.

Tinnitus represents a particularly challenging clinical condition due to its subjective symptom profile and the absence of robust objective biomarkers. Machine learning-based approaches integrating high-frequency audiometry have demonstrated promising classification performance in selected datasets [28,29], while neurophysiological and EEG-based models have been explored for objective assessment and treatment-response prediction [30,31,32,33]. Nevertheless, most studies rely on relatively small cohorts and cross-sectional designs, and standardized validation frameworks are lacking. Future progress will depend on harmonized data acquisition protocols and externally validated predictive models capable of supporting individualized management strategies rather than solely retrospective classification.

Hearing aid fitting and personalization have similarly emerged as active translational research areas. Predictive modeling using clinical fitting datasets has been investigated to estimate user benefit and optimize parameter selection [34,35], while neural network-based and adaptive optimization approaches have demonstrated promising performance in controlled or challenge-based evaluation settings [36,37,38,39]. Despite encouraging technical results, evidence demonstrating superiority over established evidence-based fitting protocols in real-world longitudinal settings remains limited. Integration into clinical workflows will require prospective outcome trials, interoperability with commercial fitting software, and transparency in algorithmic decision pathways to ensure clinician trust and patient safety.

Beyond clinical applications, the development of AI/ML research in audiology has also been influenced by funding priorities, policy frameworks, and ethical considerations. Major public research programs in Europe and the United States—such as Horizon Europe and the NIDCD Strategic Plan—have emphasized translational impact, interdisciplinary collaboration, and clinical validation in emerging health technologies [40,41].

In parallel, international governance discussions increasingly highlight the importance of explainability, reliability, and equitable deployment of artificial intelligence in healthcare systems [42,43,44]. Ethical and regulatory analyses stress that AI-based decision-support tools must align with patient safety standards, transparency requirements, and health equity principles [42,43].

These policy and governance considerations underscore that technological innovation in audiology must be accompanied by rigorous validation pathways and responsible implementation strategies.

A substantial proportion of AI/ML studies in audiology are now published in high-visibility open-access journals, reflecting the broader expansion and dissemination of artificial intelligence research in medicine [18,19]. However, high article processing charges remain a significant barrier for researchers in low- and middle-income regions, potentially reinforcing global inequities in scientific participation [45,46]. Sustainable funding mechanisms and transparent publishing practices will therefore be essential to ensure equitable and reproducible advancement of AI-driven audiology research.

Compared with earlier mapping and bibliometric reports, the present scoping review places greater focus on the translational context by explicitly relating thematic evolution to diagnostic and rehabilitative domains. By integrating bibliometric mapping within a scoping review framework, this study outlines the structural development of AI/ML research in audiology and highlights its gradual orientation toward clinically relevant applications, while acknowledging that most published studies remain at proof-of-concept or internally validated stages

This study has several limitations that should be considered when interpreting the findings. First, the analysis was restricted to publications indexed in the Web of Science Core Collection (SCI-Expanded). Web of Science was intentionally selected because it provides standardized citation metadata, reference linkage, and institutional indexing structures that are essential for reliable bibliometric and network-based analyses. This methodological consistency strengthens citation tracking and collaboration mapping. However, studies indexed exclusively in other databases (e.g., Scopus, IEEE Xplore, or PubMed) may not have been captured. Accordingly, the findings should be interpreted as reflecting the WoS-indexed segment of the literature rather than the entirety of AI/ML research in audiology.

Second, the search strategy relied on predefined combinations of AI- and audiology-related keywords. Although the query was intentionally broad, studies using modality-specific terminology (e.g., otoacoustic emissions, auditory brainstem response, electrically evoked compound action potentials) without explicit AI descriptors may have been underrepresented. This may have influenced the relative prominence of certain thematic clusters or emerging trends.

Third, translational categorization was based on reported study design characteristics and validation descriptions. As such, classification may not fully reflect methodological rigor, clinical maturity, or real-world implementation readiness.

Despite these limitations, integrating a structured scoping review methodology with complementary bibliometric and thematic mapping provides a systematic and transparent overview of research evolution, collaboration patterns, and emerging clinical directions within the WoS-indexed literature.

## 5. Conclusions

This scoping review provides a comprehensive overview of AI and ML applications in audiology and hearing disorders over the past three decades, contextualized through bibliometric and thematic analyses. The marked increase in publications after 2020 reflects accelerating research activity and expanding interdisciplinary engagement in this domain.

Current applications are primarily concentrated in diagnostic and screening contexts, including hearing loss detection, noise-induced impairment prediction, cochlear implant optimization, tinnitus modeling, and hearing aid personalization. Emerging themes such as tele-audiology and high-frequency audiometry indicate ongoing diversification aimed at improving accessibility and clinical decision support.

However, most studies remain at proof-of-concept or internally validated stages, underscoring the need for external validation, prospective clinical evaluation, methodological transparency, and alignment with ethical and regulatory frameworks. Strengthening real-world validation and standardized reporting will be essential to translate algorithmic innovation into sustainable clinical implementation.

Overall, AI and ML research in audiology is progressively transitioning from exploratory methodological development toward clinically oriented investigation. Continued emphasis on validation, reproducibility, and responsible deployment will determine the extent to which these technologies achieve durable integration into routine audiological practice.

## Figures and Tables

**Table 1:** Top 10 most productive countries by publication type (articles and reviews).

Rank	Country	Articles (n)	Reviews (n)	Total (n)
1	China	31	3	34
2	USA	19	1	20
3	South Korea	12	2	14
4	Germany	6	2	8
5	Turkey	5	1	6
6	UK	5	1	6
7	Australia	3	1	4
8	Denmark	3	0	3
9	Taiwan	3	0	3
10	Canada	2	1	3

**Table 2:** Top 10 journals publishing on AI/ML in audiology and hearing disorders. Bradford’s law zones: Zone 1 = 9 journals/43 publications; Zone 2 = 29 journals/42 publications; Zone 3 = 42 journals/42 publications.

Rank	Journal	Publications (n)	Bradford Zone
1	*Scientific Reports*	8	1
2	*Hearing Research*	6	1
3	*Frontiers in Neuroscience*	6	1
4	*Otolaryngology–Head and Neck Surgery*	5	1
5	*Ear and Hearing*	4	1
6	*Applied Sciences–Basel*	4	1
7	*Diagnostics*	3	1
8	*PLOS ONE*	3	1
9	*Frontiers in Neurology*	3	1
10	*Journal of Clinical Medicine*	3	2

**Table 3:** Top 10 keywords (Article/Review; Author Keywords and Keywords Plus).

Article—Author Keywords	Freq.	Article—Keywords Plus	Freq.	Review—Author Keywords	Freq.	Review—Keywords Plus	Freq.
Machine Learning (ML)	31	Classification	15	Artificial Intelligence	9	Classification	4
Deep Learning	22	Diagnosis	12	Machine Learning	8	Diagnosis	4
Hearing Loss	17	Hearing Loss	10	Deep Learning	4	Neural Networks	3
Artificial Intelligence (AI)	11	Noise	6	Otorhinolaryngology	2	Noise	3
Cochlear Implant	10	Recognition	6	Otology	2	Sensorineural Hearing Loss	3
Hearing Aids	7	Prevalence	6	Audiology	2	Hearing Loss	3
Sensorineural Hearing Loss	5	Children	5	Remote Care	2	Children	2
Audiogram	5	Adults	5	Mobile Phone	2	Management	2
Noise-induced Hearing Loss	5	Impairment	5	Cochlear Implant	2	Prediction	2
Audiology	4	Risk	5	Tinnitus	2	System	2

**Table 4:** Distribution of AI/ML applications in audiology according to clinical domain, methodological approach, and reported validation stage (1995–2025).

Clinical Domain	Dominant AI Approach	Most-Frequently Reported Validation Stage	Records (n)
Hearing Loss Diagnosis/ Screening	Deep Learning; Machine Learning	Predominantly Proof-of-Concept	46
Cochlear Implant Applications	Deep Learning; Machine Learning	Mainly Proof-of-Concept with Limited Internal Validation	18
Noise-Induced Hearing Loss Prediction	Machine Learning	Proof-of-Concept and Internal Validation	14
Otologic Disease Detection	Deep Learning	Proof-of-Concept and Internal Validation	12
Hearing Aid Fitting and Personalization	Deep Learning; Machine Learning	Predominantly Proof-of-Concept	9
Tinnitus Assessment and Monitoring	Machine Learning	Early-stage/Proof-of-Concept	6
Tele-Audiology and Remote Care	Machine Learning	Early-stage Validation	5
Other Specialized Audiological Applications	Mixed Approaches	Variable	17

## Data Availability

The original data presented in the study will be openly available in the Zenodo repository. The corresponding DOI will be provided at that time.

## Supplementary Materials

The following supporting information can be downloaded at: https://www.mdpi.com/article/10.3390/audiolres16020029/s1, Figure S1: PRISMA flow diagram summarizing the identification, screening, eligibility, and inclusion of studies (1995–2025) in accordance with PRISMA 2020 guidelines; Figure S2: Top 10 most productive countries in AI/ML-related audiology research (1995–2025). Bar lengths represent total publication counts; numbers on the bars denote total citations; Figure S3: Temporal distribution of top author keywords in AI/ML and audiology research. Keyword frequency was analyzed using Biblioshiny (R 4.3.1), and terms were colored according to year of occurrence; Figure S4: Co-authorship network of AI/ML publications in audiology (1995–2025). Generated using VOSviewer v1.6.19 (full counting method; minimum 3 documents per author; minimum link strength = 2). Node size represents the number of publications, link thickness indicates co-authorship strength, and colors denote collaboration clusters; Figure S5: Institutional collaboration network of AI/ML studies in audiology (1995–2025). Constructed in VOSviewer v1.6.19 (full counting method; minimum 3 documents per institution; minimum link strength = 2). Node size represents institutional publication count, link thickness shows collaboration strength, and colors denote institutional clusters; Figure S6: Country collaboration network of AI/ML publications in audiology (1995–2025). Created using VOSviewer v1.6.19 (full counting method; minimum 3 documents per country; minimum link strength = 2). Node size represents national publication output, link thickness indicates collaboration intensity, and colors denote geographic clusters; Figure S7: Top-cited references in AI/ML-related audiology research (1995–2025). Bar lengths correspond to total citation counts per reference; ranked in descending order; Figure S8: Hierarchical clustering dendrogram of co-cited references in AI/ML and audiology literature (1995–2025). Generated in Biblioshiny (R 4.3.1) using Ward’s linkage on co-citation distances, showing major thematic clusters; Figure S9: Co-occurrence network of Keywords Plus in AI/ML and audiology (1995–2025). Generated with VOSviewer v1.6.19 (full counting method; minimum occurrence = 3; minimum link strength = 2). Node size reflects keyword frequency, link thickness indicates co-occurrence strength, and colors denote thematic clusters; Table S1: Web of Science search strategy used to identify AI/ML studies in audiology and hearing disorders; Table S2: Annual publication distribution by article and review type (1995–2025); Table S3: Country-level productivity of AI/ML research articles (1995–2025); Table S4: Country-level productivity of review articles (1995–2025); Table S5: Most productive institutions (2007–2025); Table S6: Countries ranked by citation impact; Table S7: Institutions ranked by citation impact; Table S8: Author-level citation impact (top-ranked authors); Table S9: Article-level co-citation clusters; Table S10: Top 15 most-cited references; Table S11: Co-citation metrics of cited articles; Table S12: Most frequently co-cited journals; Table S13: Co-citation metrics of authors; Table S14: Article author keywords: co-occurrence analysis; Table S15: Article Keywords Plus: co-occurrence analysis; Table S16: Review author keywords: co-occurrence analysis; Table S17: Review Keywords Plus: co-occurrence analysis; Table S18: Top funding agencies; Table S19: Open access status of publications.

## Abbreviations

The following abbreviations are used in this manuscript:

AI	Artificial Intelligence
ML	Machine Learning
WoS	Web of Science
SCI-E	Science Citation Index Expanded
PRISMA-ScR	Preferred Reporting Items for Systematic Reviews and Meta-Analyses Extension for Scoping Reviews
DT	Doubling Time
APC	Article Processing Charges
WHO	World Health Organization

## References

[1] Gulshan V., Peng L., Coram M., Stumpe M.C., Wu D., Narayanaswamy A., Venugopalan S., Widner K., Madams T., Cuadros J. (2016). Development and validation of a deep learning algorithm for detection of diabetic retinopathy in retinal fundus photographs. JAMA.

[2] Esteva A., Kuprel B., Novoa R.A., Ko J., Swetter S.M., Blau H.M., Thrun S. (2017). Dermatologist-level classification of skin cancer with deep neural networks. Nature.

[3] Viscaino M., Maass J.C., Delano P.H., Torrente M., Stott C., Cheein F.A. (2020). Computer-aided diagnosis of external and middle ear conditions: A machine learning approach. PLoS ONE.

[4] Arias-Vergara T., Klumpp P., Vasquez-Correa J.C., Nöth E., Orozco-Arroyave J.R., Schuster M. (2021). Multi-channel spectrograms for speech processing applications using deep learning methods. Pattern Anal. Appl..

[5] Wasmann J.W., Pragt L., Eikelboom R., Swanepoel D.W. (2022). Digital approaches to automated and machine learning assessments of hearing: Scoping review. J. Med. Internet Res..

[6] Zhao Y., Li J., Zhang M., Lu Y., Xie H., Tian Y., Qiu W. (2019). Machine learning models for hearing impairment prediction in workers exposed to complex industrial noise: A pilot study. Ear Hear..

[7] Botros A., van Dijk B., Killian M. (2007). AutoNRT™: An automated system that measures ECAP thresholds with the Nucleus® Freedom™ cochlear implant via machine intelligence. Artif. Intell. Med..

[8] D’Onofrio K.L., Zeng F.G. (2021). Tele-audiology: Current state and future directions. Front. Digit. Health.

[9] World Health Organization (2021). World Report on Hearing.

[10] Heutink F., Koch V., Verbist B., van der Woude W.J., Mylanus E., Huinck W., Sechopoulos I., Caballo M. (2020). Multi-scale deep learning framework for cochlea localization, segmentation and analysis on clinical ultra-high-resolution CT images. Comput. Methods Programs Biomed..

[11] Wang Y.M., Li Y., Cheng Y.S., He Z.Y., Yang J.M., Xu J.H., Chi Z.C., Chi F.L., Ren D.D. (2020). Deep learning in automated region proposal and diagnosis of chronic otitis media based on computed tomography. Ear Hear..

[12] Chen Y., Yang M., Chen X., Liu B., Wang H., Wang S. (2018). Sensorineural hearing loss detection via discrete wavelet transform and machine learning methods. Multimed. Tools Appl..

[13] You E., Lin V., Mijovic T., Eskander A., Crowson M.G. (2020). Artificial intelligence applications in otology: A state-of-the-art review. Otolaryngol. Head Neck Surg..

[14] Frosolini A., Franz L., Caragli V., Genovese E., de Filippis C., Marioni G. (2024). Artificial intelligence in audiology: A scoping review of current applications and future directions. Sensors.

[15] Donthu N., Kumar S., Mukherjee D., Pandey N., Lim W.M. (2021). How to conduct a bibliometric analysis: An overview and guidelines. J. Bus. Res..

[16] Callon M., Courtial J.P., Laville F. (1991). Co-word analysis as a tool for describing the network of interactions between basic and technological research: The case of polymer chemistry. Scientometrics.

[17] Cobo M.J., López-Herrera A.G., Herrera-Viedma E., Herrera F. (2011). Science mapping software tools: Review, analysis, and cooperative study among tools. J. Am. Soc. Inf. Sci. Technol..

[18] Tran B.X., Vu G.T., Ha G.H., Vuong Q.-H., Ho M.-T., Vuong T.-T., La V.-P., Ho M.-T., Nghiem K.-C.P., Nguyen H.L.T. (2019). Global evolution of research in artificial intelligence in health and medicine: A bibliometric study. J. Clin. Med..

[19] Lin M., Lin L., Lin L., Lin Z., Yan X. (2025). A bibliometric analysis of the advance of artificial intelligence in medicine. Front. Med..

[20] Demir E., Uğurlu B.N., Uğurlu G.A., Aydoğdu G. (2025). Artificial intelligence in otorhinolaryngology: Current trends and application areas. Eur. Arch. Otorhinolaryngol..

[21] van Raan A.F.J. (2005). Statistical properties of bibliometric indicators: Research group indicator distributions and correlations. J. Am. Soc. Inf. Sci. Technol..

[22] Chen C., Ibekwe-SanJuan F., Hou J. (2010). The structure and dynamics of cocitation clusters: A mutiple-perspective cocitation analysis. J. Am. Soc. Inf. Sci. Technol..

[23] Ding Y., Yan E., Frazier S., Cronin B. (2011). Scientific collaboration and endorsement: Network analysis of coauthorship and citation networks. J. Informetr..

[24] Katz J.S., Martin B.R. (1997). What is research collaboration?. Res. Policy.

[25] Abramo G., D’Angelo C.A., Solazzi M. (2011). The relationship between scientists’ research performance and the degree of internationalization of their research. Scientometrics.

[26] Bradford S.C. (1976). Sources of information on specific subjects. J. Inf. Sci..

[27] Manchaiah V., Dockens A.L., Ahmadi T. (2019). Peer-reviewed publication trends in Ear and Hearing: A 40-year bibliometric analysis. Ear Hear..

[28] Sadegh-Zadeh S.-A., Mamalo A.S., Kavianpour K., Atashbar H., Heidari E., Hajizadeh R., Roshani A.S., Habibzadeh S., Saadat S., Behmanesh M. (2024). Artificial intelligence approaches for tinnitus diagnosis: Leveraging high-frequency audiometry data for enhanced clinical predictions. Front. Artif. Intell..

[29] Jafari Z., Harari R.E., Hole G., Kolb B.E., Mohajerani M.H. (2025). Machine learning models can predict tinnitus and noise-induced hearing loss. Ear Hear..

[30] Kim J., Lim K.H., Kim E., Kim S., Kim H.J., Lee Y.H., Kim S., Choi J. (2025). Machine learning-based diagnosis of chronic subjective tinnitus with altered cognitive function: An event-related potential study. Ear Hear..

[31] Doborjeh M., Liu X., Doborjeh Z., Shen Y., Searchfield G., Sanders P., Wang G.Y., Sumich A., Yan W.Q. (2023). Prediction of tinnitus treatment outcomes based on EEG sensors and TFI score using deep learning. Sensors.

[32] Adcock K.S., Byczynski G., Meade E., Leong S.L., Gault R., Lim H., Vanneste S. (2024). Feasibility of deep learning to predict tinnitus patient outcomes. Intell.-Based Med..

[33] Shoushtarian M., Alizadehsani R., Khosravi A., Acevedo N., McKay C.M., Nahavandi S., Fallon J.B. (2020). Objective measurement of tinnitus using functional near-infrared spectroscopy and machine learning. PLoS ONE.

[34] Roger P., Lespargot T., Boiteux C., Bailly-Masson E., Auberger F., Mouysset S., Fraysse B., Boiteux C. (2025). Predicting hearing aid outcomes using machine learning. Audiol. Neurotol..

[35] Mondol S.R., Kim H.J., Kim K.S., Lee S. (2022). Machine learning-based hearing aid fitting personalization using clinical fitting data. J. Healthc. Eng..

[36] Tasnim N.Z., Ni A., Lobarinas E., Kehtarnavaz N. (2024). A review of machine learning approaches for the personalization of amplification in hearing aids. Sensors.

[37] Balling L.W., Mølgaard L.L., Townend O., Nielsen J.B.B. (2021). The collaboration between hearing aid users and artificial intelligence to optimize sound. Semin. Hear..

[38] Fabry D.A., Bhowmik A.K. (2021). Improving speech understanding and monitoring health with hearing aids using artificial intelligence and embedded sensors. Semin. Hear..

[39] Graetzer S., Barker J., Cox T.J., Akeroyd M., Culling J.F., Naylor G., Porter E., Muñoz R.V. (2021). Clarity-2021 challenges: Machine learning challenges for advancing hearing aid processing. Proc. Interspeech.

[40] European Commission (2021). Horizon Europe: The EU Framework Programme for Research and Innovation 2021–2027.

[41] National Institute on Deafness and Other Communication Disorders (NIDCD) (2023). NIDCD Strategic Plan 2023–2027: Advancing the Science of Communication to Improve Lives.

[42] Goktas P., Grzybowski A. (2025). Shaping the future of healthcare: Ethical clinical challenges and pathways to trustworthy AI. J. Clin. Med..

[43] Johnson K.B. (2025). Pursuing equity with artificial intelligence in health care. JAMA Health Forum.

[44] World Health Organization (2025). Four strategic priorities of the global governance framework for health AI. npj Digit. Med..

[45] Solomon D.J., Björk B.C. (2016). Article processing charges for open access publication—The situation for research intensive universities in the USA and Canada. PeerJ.

[46] Khoo S.Y.S. (2019). Article processing charge hyperinflation and price insensitivity: An open access sequel to the serials crisis. LIBER Q..

