# Supplementary material for: Artificial Intelligence and Machine Learning in Audiology and Hearing Disorders: A Scoping Review with Bibliometric and Thematic Mapping (1995–2025)
Source: Audiol Res. 2026 Feb 24;16(2):29. doi: 10.3390/audiolres16020029 (PMC13010648; doi:10.3390/audiolres16020029)
Supplement: Supplementary file 1 [file audiolres-16-00029-s001.zip › Supplementary_information.pdf]

Supplementary Information

Artificial Intelligence and Machine Learning in Audiology: A Global Bibliometric and Network Analysis (1995–2025)

Ceren Aksoy Koçak

Table S1. Web of Science search strategy used to identify AI/ML studies in audiology and hearing disorders.

Search Field	Query (TS)
Topic	("artificial intelligence" OR "machine learning" OR "deep learning" OR "neural network*" OR "algorithm*" OR "supervised learning" OR "unsupervised learning" OR "computer-aided" OR "automatic*" OR "AI" OR "ML") AND ("audiology" OR "hearing loss" OR "hearing disorder*" OR "cochlear implant*" OR "tinnitus" OR "otology" OR "hearing aid*" OR "auditory" OR "hearing test*" OR "pure-tone" OR "speech audiometry")

Notes:

• Search performed in WoS Core Collection (SCI-E) on 5 August 2025.

• Filters: English language; Article and Review; no year/journal restriction.

• Wildcard asterisk (*) used for word derivatives.

Table S2. Annual publication distribution by article and review type (1995–2025).

Year	Articles	Reviews	Total
1995	1	0	1
2007	1	0	1
2008	1	0	1
2012	1	0	1
2013	2	0	2
2018	1	0	1
2019	3	0	3
2020	6	0	6
2021	8	1	9
2022	10	3	13
2023	18	5	23
2024	28	4	32
2025	31	3	34

Table S3. Country-level productivity of AI/ML research articles (1995–2025).

Rank	Country	Number of Articles (n)	Percentage (%)
1	China	31	27.9
2	USA	19	17.1
3	South Korea	12	10.8
4	Germany	6	5.4
5	Turkey	5	4.5
6	UK	5	4.5

Rank	Country	Number of Articles (n)	Percentage (%)
7	Australia	3	2.7
8	Denmark	3	2.7
9	Taiwan	3	2.7
10	Canada	2	1.8
11	Netherlands	2	1.8
12	Japan	2	1.8
13	India	2	1.8
14	Israel	1	0.9
15	Portugal	1	0.9
16	Switzerland	1	0.9
17	Italy	1	0.9
18	Greece	1	0.9
19	Egypt	1	0.9
20	South Africa	1	0.9
21	Saudi Arabia	1	0.9
22	Colombia	1	0.9
23	France	1	0.9
24	Poland	1	0.9
25	Finland	1	0.9
26	Malaysia	1	0.9
27	Chile	1	0.9

Rank	Country	Number of Articles (n)	Percentage (%)
28	Russia	1	0.9
29	Thailand	1	0.9

Table S4. Country-level productivity of review articles (1995–2025).

Rank	Country	Reviews (n)
1	China	3
2	South Korea	1
3	Canada	1
4	Iran	1
5	Malaysia	1
6	Netherlands	1
7	Australia	1
8	France	1
9	Germany	1
10	United Kingdom	1
11	Bangladesh	1
12	Turkey	1
13	United States	1
14	Japan	1

Table S5. Most productive institutions (2007–2025).

Rank	Institution	Country	Number of Articles
1	Carl von Ossietzky Universität Oldenburg	Germany	4
2	Soonchunhyang University	South Korea	3
3	Peking University	China	3
4	Chongqing University	China	3
5	University of Iowa	USA	3
6	Hanyang University	South Korea	2
7	Technical University of Denmark	Denmark	2
8	Fudan University	China	2
9	University System of Ohio	USA	2
10	Firat University	Turkey	2
11	Vanderbilt University	USA	2
12	Shanghai Jiao Tong University	China	2
13	Korea University	South Korea	2

Table S6. Countries ranked by citation impact.

Country	Publications	Total Citations	Mean Citations	H-index
Chile	1	57	57.0	1
South Korea	6	54	9.0	2
United Kingdom	7	48	6.86	4
China	5	48	9.6	2

Canada	2	47	23.5	2
Australia	2	40	20.0	1
Denmark	2	20	10.0	1
Hong Kong	2	16	8.0	2
Malaysia	2	8	4.0	2
Egypt	1	4	4.0	1

Table S7. Institutions ranked by citation impact.

Institution	Publications	Total Citations	Mean Citations	H-index
Radboud University Nijmegen	4	176	44.0	4
University of Erlangen Nuremberg	3	115	38.3	3
University of Iowa	3	90	30.0	3
Cochlear	1	76	76.0	1
Universidad de Antioquia	1	68	68.0	1
University of Munich	1	68	68.0	1
Leiden University (incl. LUMC)	1	61	61.0	1
Vanderbilt University	3	59	19.7	2

Table S8. Author-level citation impact (top-ranked authors).

Rank	Author	Publications	Total Citations	Mean Citations	h-index
1	Arias-Vergara, T	1	66	66.0	1
2	Botros, A	1	63	63.0	1
3	Heutink, F	1	56	56.0	1
4	Viscaino, M	1	52	52.0	1
5	Wang, YM	1	47	47.0	1
6	Schilling, A	2	46	23.0	2
7	Zhao, YX	1	44	44.0	1
8	Chen, Y	1	39	39.0	1
9	Shew, M	2	37	18.5	2
10	Wasmann, JW	1	37	37.0	1
11	Hildebrand, MS	1	37	37.0	1
12	You, EI	1	35	35.0	1
13	Tama, BA	1	34	34.0	1
14	Taylor, KR	1	29	29.0	1
15	Konrad-Martin, D	1	24	24.0	1
16	Chen, FF	1	19	19.0	1
17	Margeta, J	1	19	19.0	1
18	Nisar, S	1	18	18.0	1

19	Lenatti, M	1	18	18.0	1
20	Lee, MK	1	17	17.0	1

Table S9. Article-level co-citation clusters.

Title	Authors	Source	Times Cited
Multi-channel spectrograms for speech processing applications using Deep Learning methods	Arias-Vergara, T; Klumpp, P; Vasquez-Correa, JC; Nöth, E; Orozco-Arroyave, JR; Schuster, M	Pattern Analysis and Applications	65
AutoNRT™: An automated system that measures ECAP thresholds with the Nucleus® Freedom™ cochlear implant via machine intelligence	Botros, A; van Dijk, B; Killian, M	Artificial Intelligence in Medicine	63
Multi-Scale Deep Learning framework for cochlea localization, segmentation and analysis on clinical ultra-high-resolution CT images	Heutink, F; Koch, V; Verbist, B; van der Woude, WJ; Mylanus, E; Huinck, W; Sechopoulos, I; Caballo, M	Computer Methods and Programs in Biomedicine	55

Computer-aided diagnosis of external and middle ear conditions: A Machine Learning (ML) approach	Viscaino, M; Maass, JC; Delano, PH; Torrente, M; Stott, C; Cheein, FA	PLOS ONE	51
Deep Learning in Automated Region Proposal and Diagnosis of Chronic Otitis Media Based on Computed Tomography	Wang, YM; Li, YK; Cheng, YS; He, ZY; Yang, JM; Xu, JH; Chi, ZC; Chi, FL; Ren, DD	Ear and Hearing	45
Machine Learning Models for the Hearing Impairment Prediction in Workers Exposed to Complex Industrial Noise: A Pilot Study	Zhao, YX; Li, JS; Zhang, MB; Lu, Y; Xie, HW; Tian, Y; Qiu, W	Ear and Hearing	44
Digital Approaches to Automated and Machine Learning Assessments of Hearing: Scoping Review	Wasmann, JW; Pragt, L; Eikelboom, R; Swanepoel, D	Journal of Medical Internet Research	40
Sensorineural hearing loss detection via discrete wavelet transform and principal component analysis combined with generalized eigenvalue proximal support vector machine and Tikhonov regularization	Chen, Y; Yang, M; Chen, XQ; Liu, B; Wang, HN; Wang, SH	Multimedia Tools and Applications	39
Audioprofile-directed screening identifies novel mutations in KCNQ4	Hildebrand, MS; Tack, D; McMordie, SJ; DeLuca, A; Hur,	Genetics in Medicine	37

causing hearing loss at the DFNA2 locus	IA; Nishimura, C; Huygen, P; Casavant, TL; Smith, RJH		
Artificial Intelligence Applications in Otology: A State of the Art Review	You, EI; Lin, V; Mijovic, T; Eskander, A; Crowson, MG	Otolaryngology–Head and Neck Surgery	37

Table S10. Top 15 most-cited references.

Rank	Reference	Citations	Category
1	Bing D, 2018, Clin Otolaryngol	15	Audiology / Otology
2	He KM, 2016, Proc CVPR IEEE (ResNet)	13	AI – Deep Learning
3	Zhao YX, 2019, Ear Hearing	12	Audiology
4	Pedregosa F, 2011, J Mach Learn Res (Scikit-learn)	12	AI – Methods
5	Cha D, 2019, EBiomedicine	9	AI in Medicine
6	Khan MA, 2020, Neural Networks	9	AI – Neural Networks
7	Breiman L, 2001, Mach Learn (Random Forests)	9	AI – ML Algorithms
8	Hochreiter S, 1997, Neural Comput (LSTM)	9	AI – Neural Networks
9	Huang G, 2017, Proc CVPR IEEE (DenseNet)	8	AI – Deep Learning
10	McKearney RM, 2019, Int J Audiol	8	Audiology

11	Simonyan K, 2015, arXiv (VGGNet)	8	AI – Deep Learning
12	Cao ZW, 2023, Laryngoscope	7	Otolaryngology
13	Park KV, 2020, Clin Exp Otorhinolaryngol	7	Otolaryngology
14	Wang YM, 2020, Ear Hearing	6	Audiology
15	Krizhevsky A, 2017, Commun ACM (AlexNet)	6	AI – Deep Learning

Table S11. Co-citation metrics of cited articles.

Reference (Author, Year, Source)	Co-citation Strength (Σ edge weight)	Co-cited Links (Degree)	CR Frequency (raw)	Unique Citing Articles
Bing D, 2018, Clin Otolaryngol	959	856	15	15
Anonymous	725	708	34	13
Graves A, 2012, Stud Comput Intell	718	658	9	9
Zhao Yx, 2019, Ear Hearing	631	582	11	11
Gates Ga, 2005, Lancet	628	624	5	5
Rusk N, 2016, Nat Methods	614	562	6	6
Pedregosa F, 2011, J Mach Learn Res	513	476	11	11

Khan Ma, 2020, Neural Networks	451	383	7	7
Conlon B, 2020, Sci Transl Med	442	441	2	2
Shekhawat Gs, 2013, J Am Acad Audiol	442	441	2	2
He Km, 2016, Proc Cvpr Ieee	438	426	10	10
Mckearney Rm, 2019, Int J Audiol	436	401	8	8
Hullfish J, 2019, Neurosci Biobehav R	400	398	2	2
Shore Se, 2016, Nat Rev Neurol	400	398	2	2
Cao Zw, 2023, Laryngoscope	397	369	7	7
Park Kv, 2020, Clin Exp Otorhinolar	395	364	7	7
Crowson Mg, 2020, Otol Neurotol	392	377	7	6
Goehring T, 2017, Hearing Res	387	357	5	5

Vaswani A, 2017, Adv Neur In	375	363	6	6
Lv Y, 2021, Int J Med Robot Comp	372	341	4	4

Table S12. Most frequently co-cited journals.

Rank	Journal	Co-citation Strength (Σ edge weight)	Co-cited Links (Degree)	CR Frequency (raw)	Unique Citing Articles
1	Ear and Hearing	2794	1301	232	73
2	Scientific Reports	2217	1124	95	52
3	Hearing Research	2110	1116	159	51
4	International Journal of Audiology	2105	1092	133	55
5	PLoS ONE	2039	1081	87	47
6	Laryngoscope	2027	1029	107	51
7	Journal of the Acoustical Society of America	1827	967	235	47

8	Otology & Neurotology	1800	921	140	45
9	Otolaryngology–Head and Neck Surgery	1669	866	89	41
10	Acta Oto-Laryngologica	1386	789	43	30

Table S13. Co-citation metrics of authors.

Author (surname, initials possible)	Co-citation Strength (Σ edge weight)	Co-cited Links (Degree)	CR Frequency (raw)	Unique Citing Articles
Anonymous	2408	2088	107	46
Crowson Mg	1207	1026	28	22
Bing D	924	799	16	16
Wang J	708	659	11	10
Graves A	664	600	11	11
Zeng Fg	646	563	14	7
Zhao Yx	631	568	13	12
Lecun Y	627	568	10	10
He Km	564	524	14	13
Gates Ga	551	535	5	5

Wilson Bs	519	472	14	10
Rusk N	515	461	6	6
Mckearney Rm	502	442	11	10
De Ridder D	498	450	6	3
Shore Se	498	450	6	3
Moore Bcj	493	451	8	6
Pedregosa F	469	426	11	11
Healy Ew	469	436	11	6
Lesica Na	465	431	7	7
World Health Organization	462	451	9	9

Table S14. Article author keywords: co-occurrence analysis.

Rank	Keyword	Cluster	Frequency / Degree
1	Deep Learning	1	22 / 9
2	Hearing Loss	1	17 / 3
3	Artificial Intelligence (AI)	1	11 / 4
4	Cochlear Implant	1	10 / 3
5	Hearing Aids	1	7 / 1
6	otitis media with effusion	1	3 / 1
7	image segmentation	1	2 / 1
8	VGG16	1	2 / 1

9	transformer	1	2 / 1
10	Machine Learning (ML)	2	31 / 10
11	Audiogram	2	5 / 1
12	Noise-induced Hearing Loss	2	5 / 1
13	Sensorineural Hearing Loss	2	5 / 1
14	Age-related Hearing Loss	2	4 / 1
15	Speech recognition	2	3 / 1
16	Meniere's disease	2	2 / 1
17	pure-tone audiometry	3	2 / 2
18	regression analysis in audiology	3	2 / 2
19	speech audiometry	3	2 / 2
20	Auditory system	4	3 / 1
21	Location awareness	4	2 / 1

Table S15. Article Keywords Plus: co-occurrence analysis.

Rank	Keyword	Cluster	Frequency / Degree
1	RECOGNITION	1	6 / 6
2	NOISE	1	6 / 2
3	PERCEPTION	1	4 / 1
4	NEURAL-NETWORKS	1	4 / 1
5	INTELLIGIBILITY	1	3 / 1
6	PREDICTION	1	3 / 1
7	HEARING-IMPAIRED LISTENERS	1	2 / 1

8	AUDIOVISUAL ASYNCHRONY DETECTION	1	2 / 1
9	CLASSIFICATION	2	15 / 5
10	HEARING-LOSS	2	10 / 1
11	PREVALENCE	2	6 / 3
12	IMPAIRMENT	2	5 / 1
13	SYSTEM	2	4 / 1
14	PERFORMANCE	2	4 / 1
15	VALIDATION	2	3 / 2
16	TYMPANOMETRY	3	5 / 4
17	CHILDREN	3	5 / 1
18	CONDUCTIVE HEARING-LOSS	3	4 / 2
19	ABSORBENCY	3	3 / 2
20	DIAGNOSIS	4	12 / 3
21	PROGNOSIS	4	2 / 1
22	HIGH-FREQUENCY AUDIOMETRY	4	2 / 1
23	RISK	5	5 / 1
24	SENSORINEURAL HEARING-LOSS	5	4 / 1

Table S16. Review author keywords: co-occurrence analysis.

Rank	Keyword	Cluster	Frequency / Degree
1	Artificial Intelligence	1	9 / 5
2	AI	1	2 / 1
3	otology	1	2 / 1

4	Deep Learning	2	4 / 3
5	Otorhinolaryngology	2	2 / 2
6	Machine Learning	3	8 / 3
7	Audiology	3	2 / 1

Table S17. Review Keywords Plus: co-occurrence analysis.

Rank	Keyword	Cluster	Frequency / Degree
1	DIAGNOSIS	1	4 / 3
2	CLASSIFICATION	1	4 / 3
3	SENSORINEURAL HEARING-LOSS	1	3 / 2
4	PROGNOSIS	1	2 / 2
5	CHILDREN	2	2 / 1
6	MANAGEMENT	2	2 / 1

Table S18. Top funding agencies.

**Funding Agency**	**Frequency**
National Key Research	5
BK21 FOUR (Fostering Outstanding Universities for Research)	3
Soonchunhyang University Research Fund	3
Ministry of Science and ICT (Republic of Korea)	3
BK21 FOUR (Research Grant)	2
Communications Technology Planning	2
Korea University Grant	2

Funding Agency	Frequency
Welfare, Republic of Korea	2
National Institute on Deafness	2
Science	2

Table S19. Open access status of publications.

Category	Publications	Percentage
Gold	53	41.7%
Not Declared	44	34.6%
Hybrid	16	12.6%
Green	11	8.7%
Bronze	3	2.4%

Figure S1. PRISMA flow diagram summarizing the identification, screening, eligibility, and inclusion of studies (1995–2025) in accordance with PRISMA 2020 guidelines.

Figure S2. Top 10 most productive countries in AI/ML-related audiology research (1995–2025). Bar lengths represent total publication counts; numbers on the bars denote total citations.

Figure S3. Temporal distribution of top author keywords in AI/ML and audiology research. Keyword frequency was analyzed using Biblioshiny (R 4.3.1), and terms were colored according to year of occurrence.

Figure S4. Co-authorship network of AI/ML publications in audiology (1995–2025). Generated using VOSviewer v1.6.19 (full counting method; minimum 3 documents per author; minimum link strength = 2). Node size represents the number of publications, link thickness indicates co-authorship strength, and colors denote collaboration clusters.

Figure S5. Institutional collaboration network of AI/ML studies in audiology (1995–2025). Constructed in VOSviewer v1.6.19 (full counting method; minimum 3 documents per institution; minimum link strength = 2). Node size represents institutional publication count, link thickness shows collaboration strength, and colors denote institutional clusters.

Figure S6. Country collaboration network of AI/ML publications in audiology (1995–2025). Created using VOSviewer v1.6.19 (full counting method; minimum 3 documents per country; minimum link strength = 2). Node size represents national publication output, link thickness indicates collaboration intensity, and colors denote geographic clusters.

Figure S7.Top-cited references in AI/ML-related audiology research (1995–2025). Bar lengths correspond to total citation counts per reference; ranked in descending order.

Figure S8. Hierarchical clustering dendrogram of co-cited references in AI/ML and audiology literature (1995–2025). Generated in Biblioshiny (R 4.3.1) using Ward's linkage on co-citation distances, showing major thematic clusters.

Figure S9. Co-occurrence network of Keywords Plus in AI/ML and audiology (1995–2025). Generated with VOSviewer v1.6.19 (full counting method; minimum occurrence = 3; minimum link strength = 2). Node size reflects keyword frequency, link thickness indicates co-occurrence strength, and colors denote thematic clusters.
