# Supplementary figures and images for: Artificial Intelligence and Machine Learning in Audiology and Hearing Disorders: A Scoping Review with Bibliometric and Thematic Mapping (1995–2025)
Source: Audiol Res. 2026 Feb 24;16(2):29. doi: 10.3390/audiolres16020029 (PMC13010648; doi:10.3390/audiolres16020029)

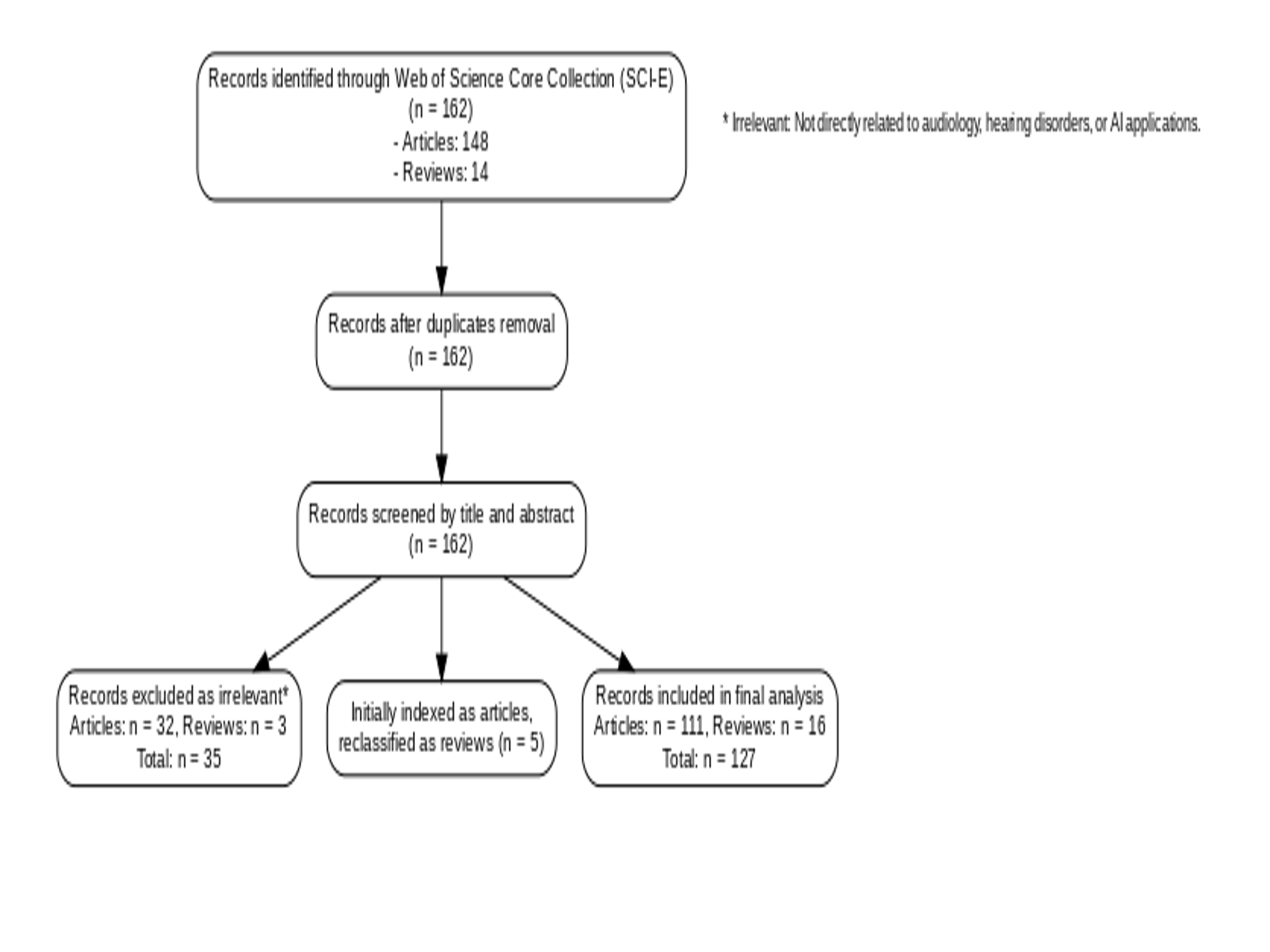

Supplement: Supplementary file 1 [file audiolres-16-00029-s001.zip › Figure S1.tiff]

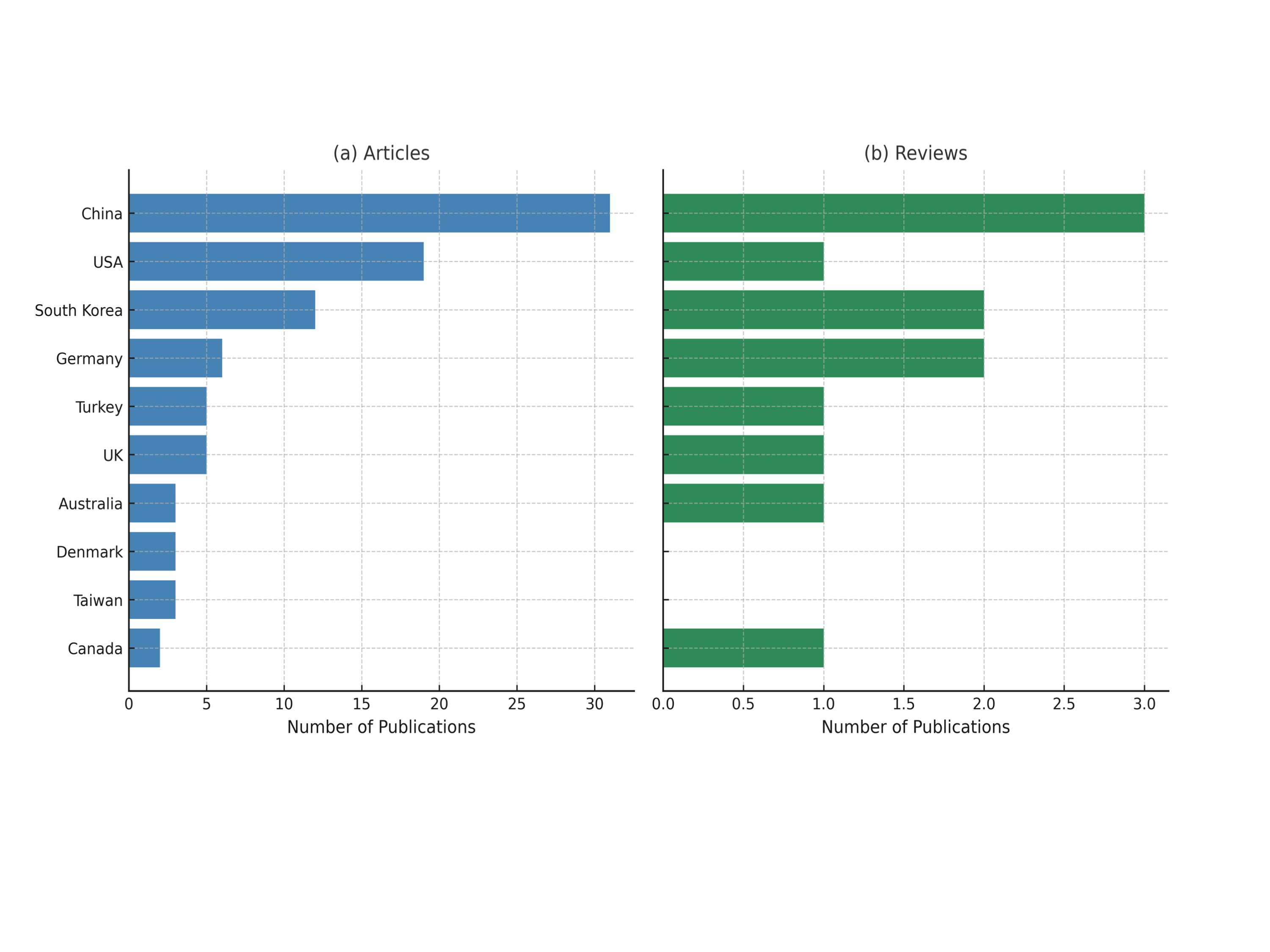

Supplement: Supplementary file 1 [file audiolres-16-00029-s001.zip › Figure S2.tiff]

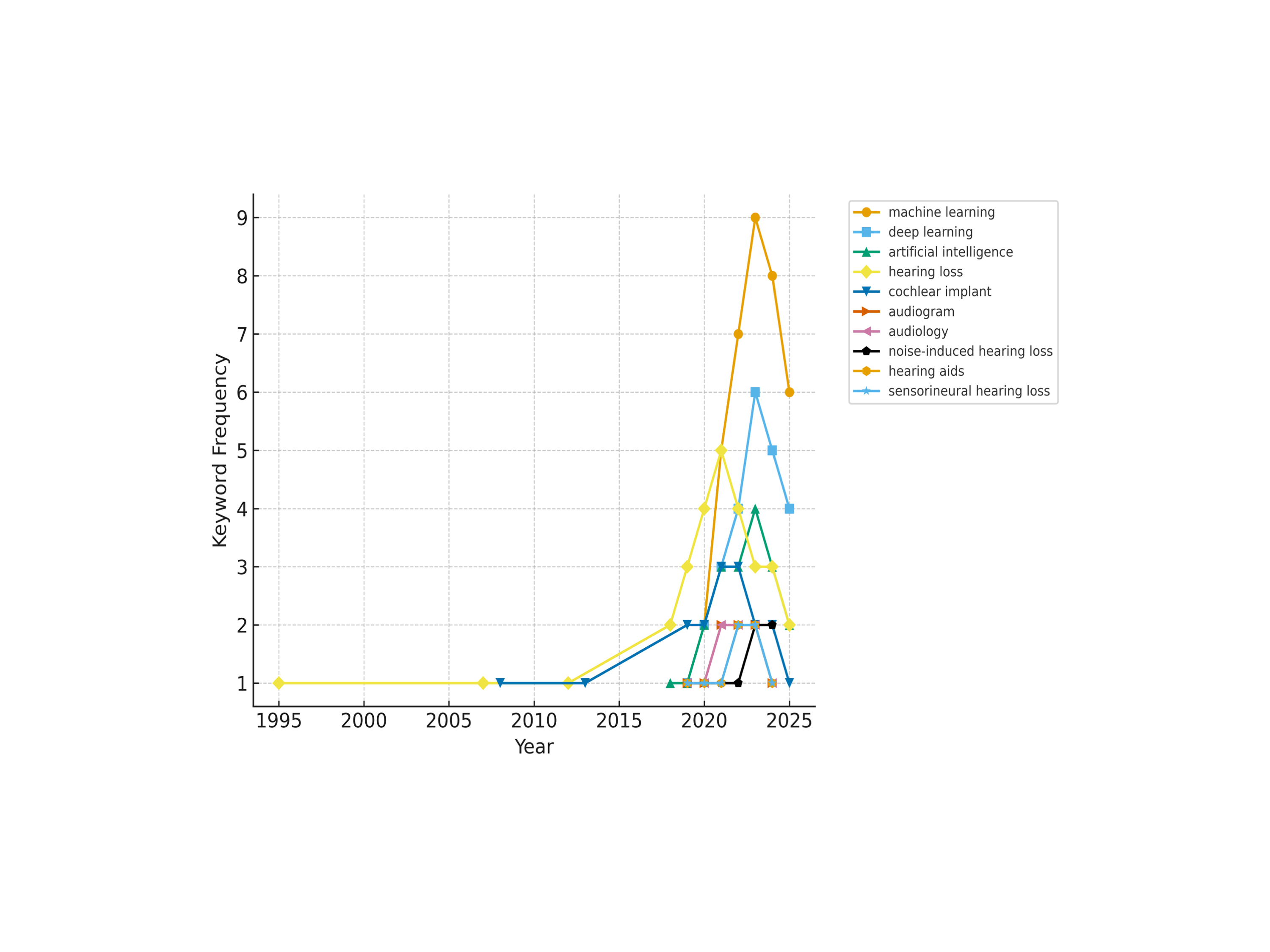

Supplement: Supplementary file 1 [file audiolres-16-00029-s001.zip › Figure S3.tiff]

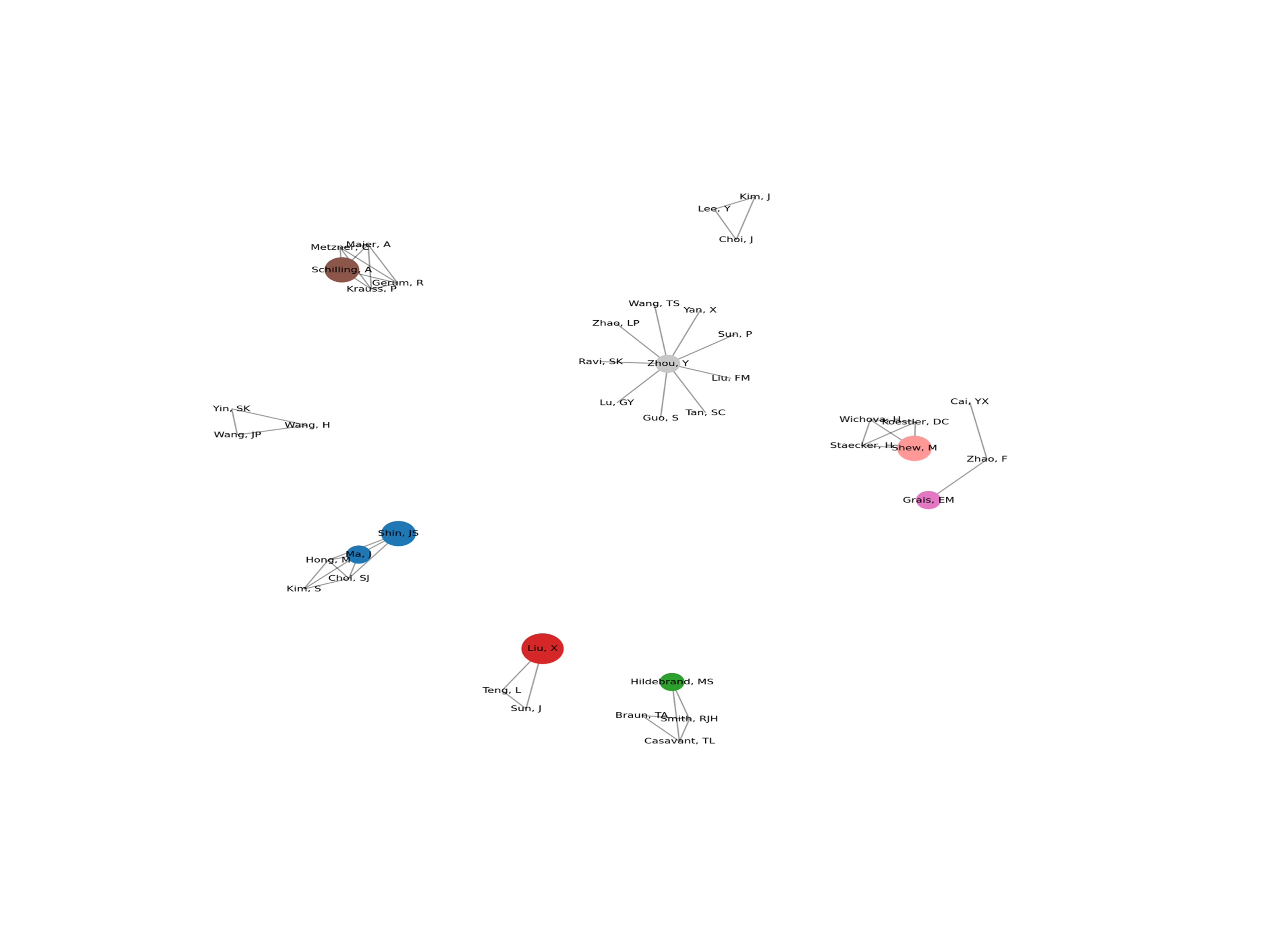

Supplement: Supplementary file 1 [file audiolres-16-00029-s001.zip › Figure S4.tiff]

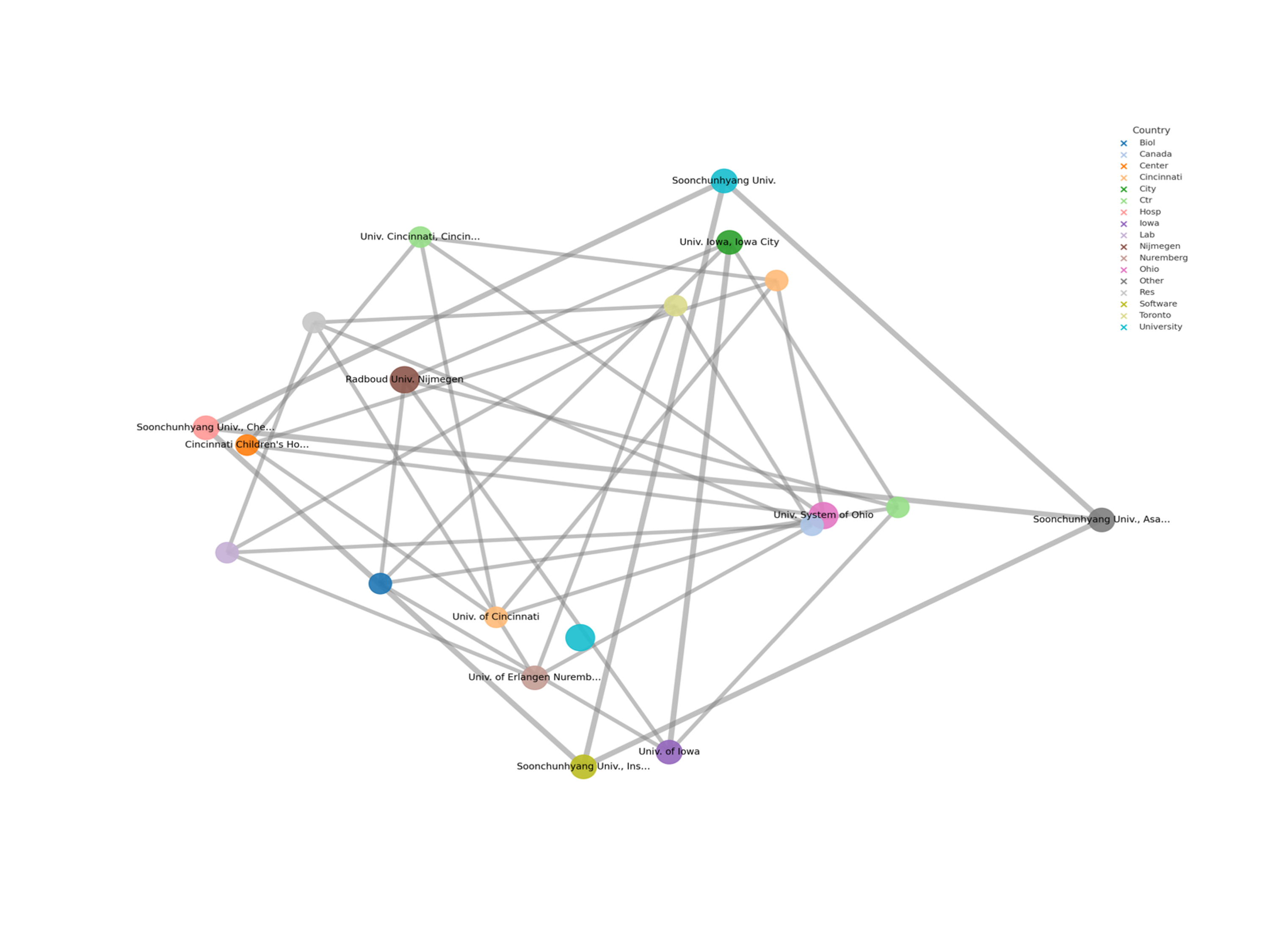

Supplement: Supplementary file 1 [file audiolres-16-00029-s001.zip › Figure S5.tiff]

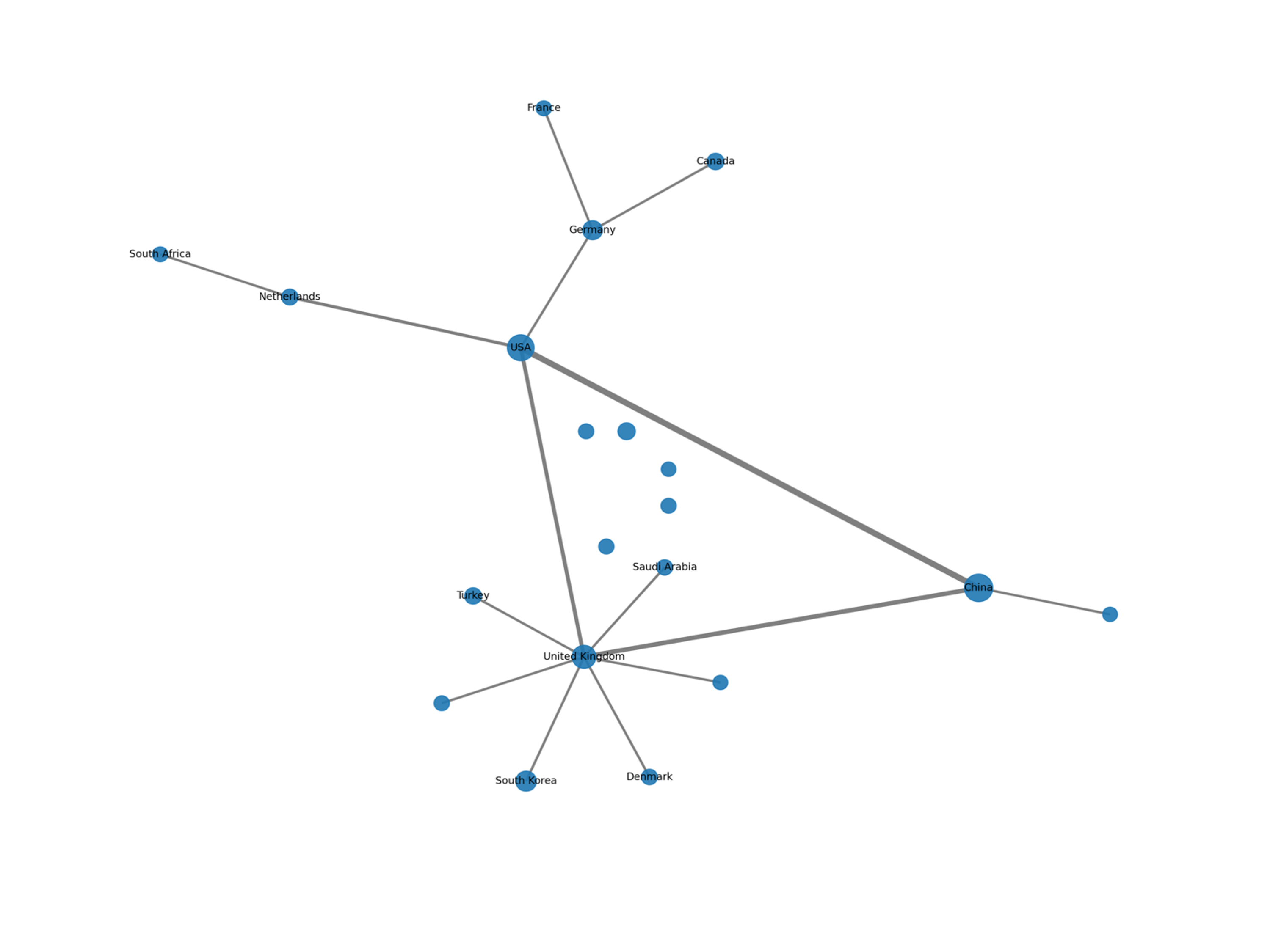

Supplement: Supplementary file 1 [file audiolres-16-00029-s001.zip › Figure S6.tiff]

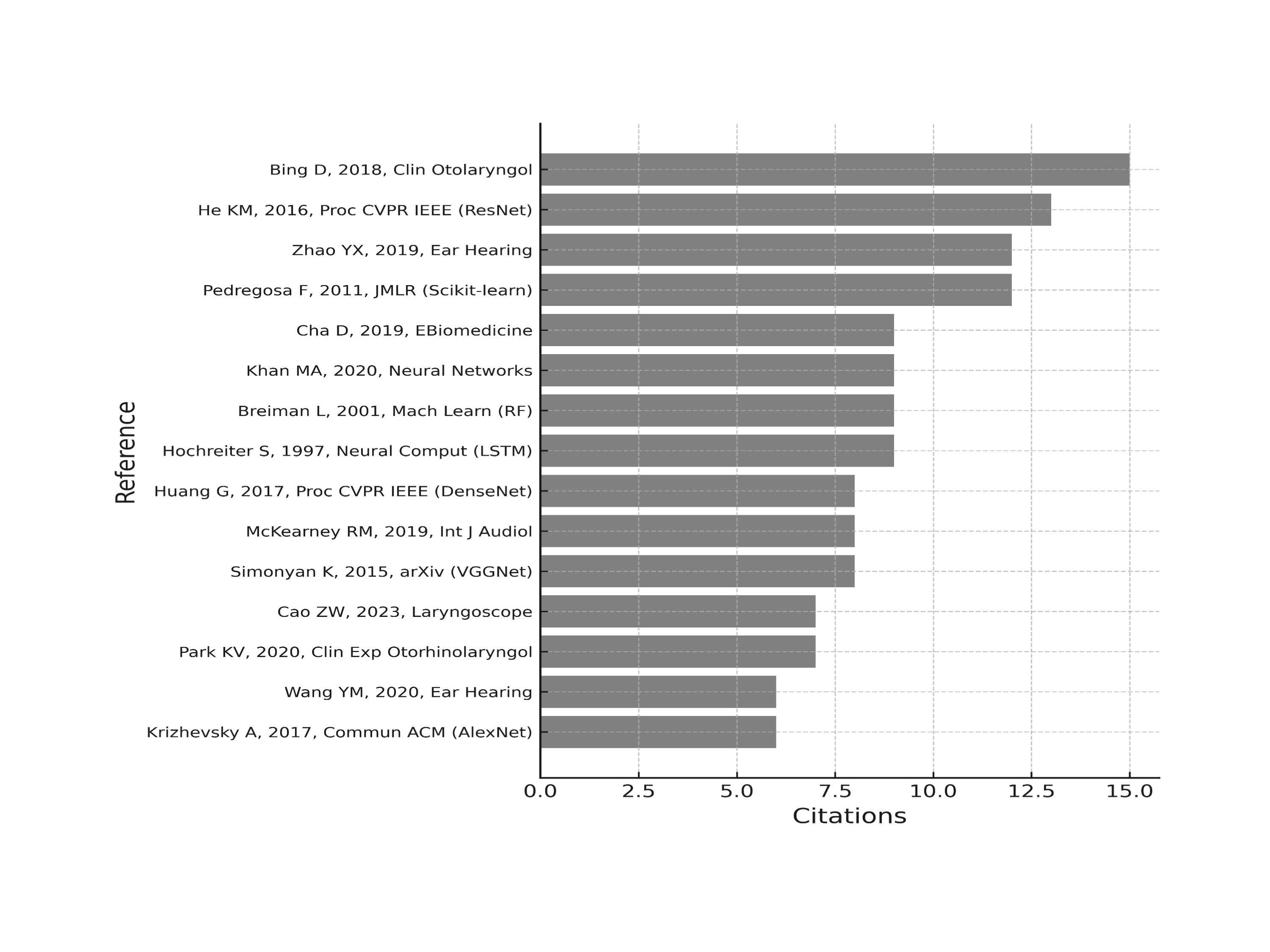

Supplement: Supplementary file 1 [file audiolres-16-00029-s001.zip › Figure S7.tiff]

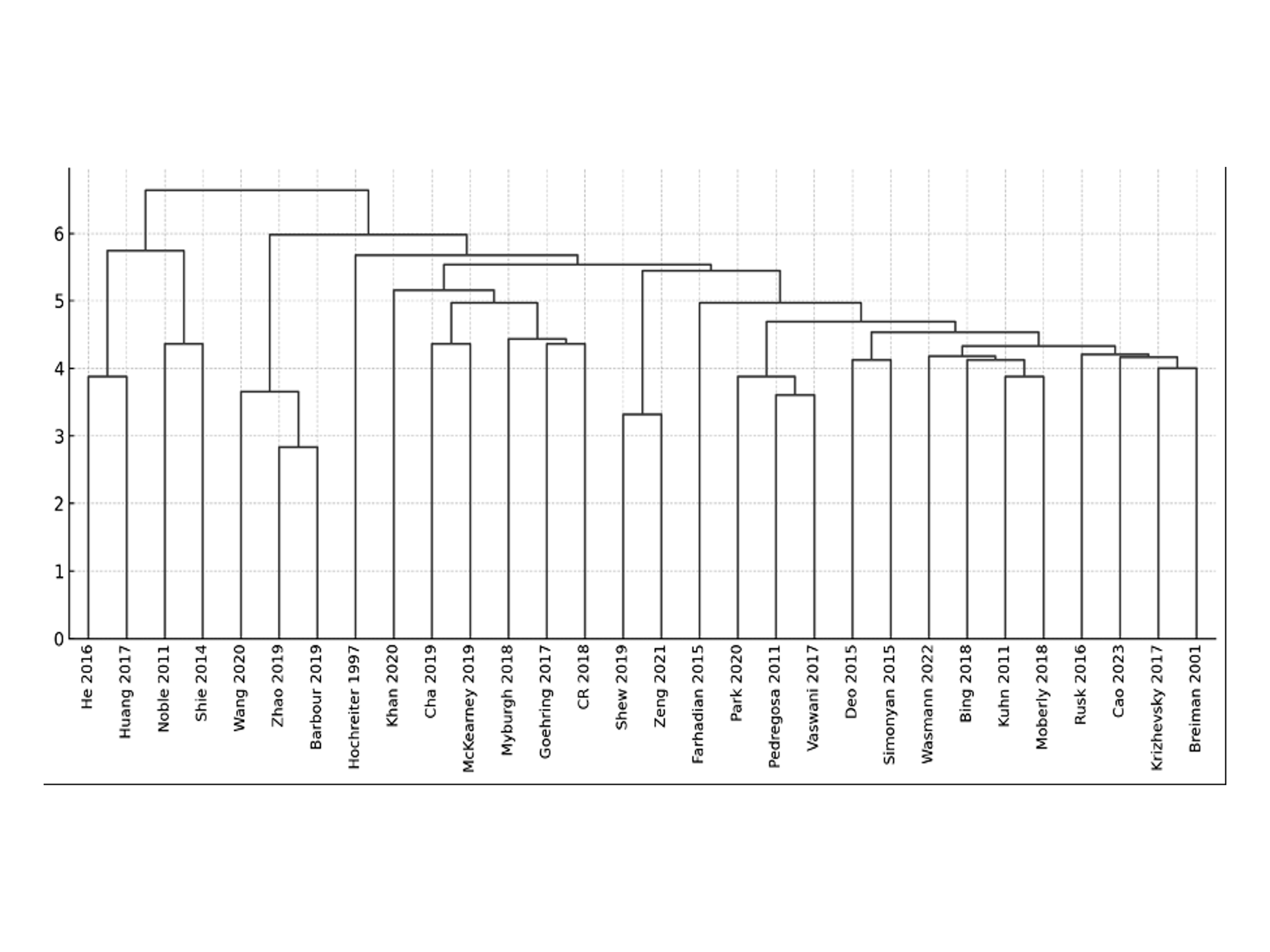

Supplement: Supplementary file 1 [file audiolres-16-00029-s001.zip › Figure S8.tiff]

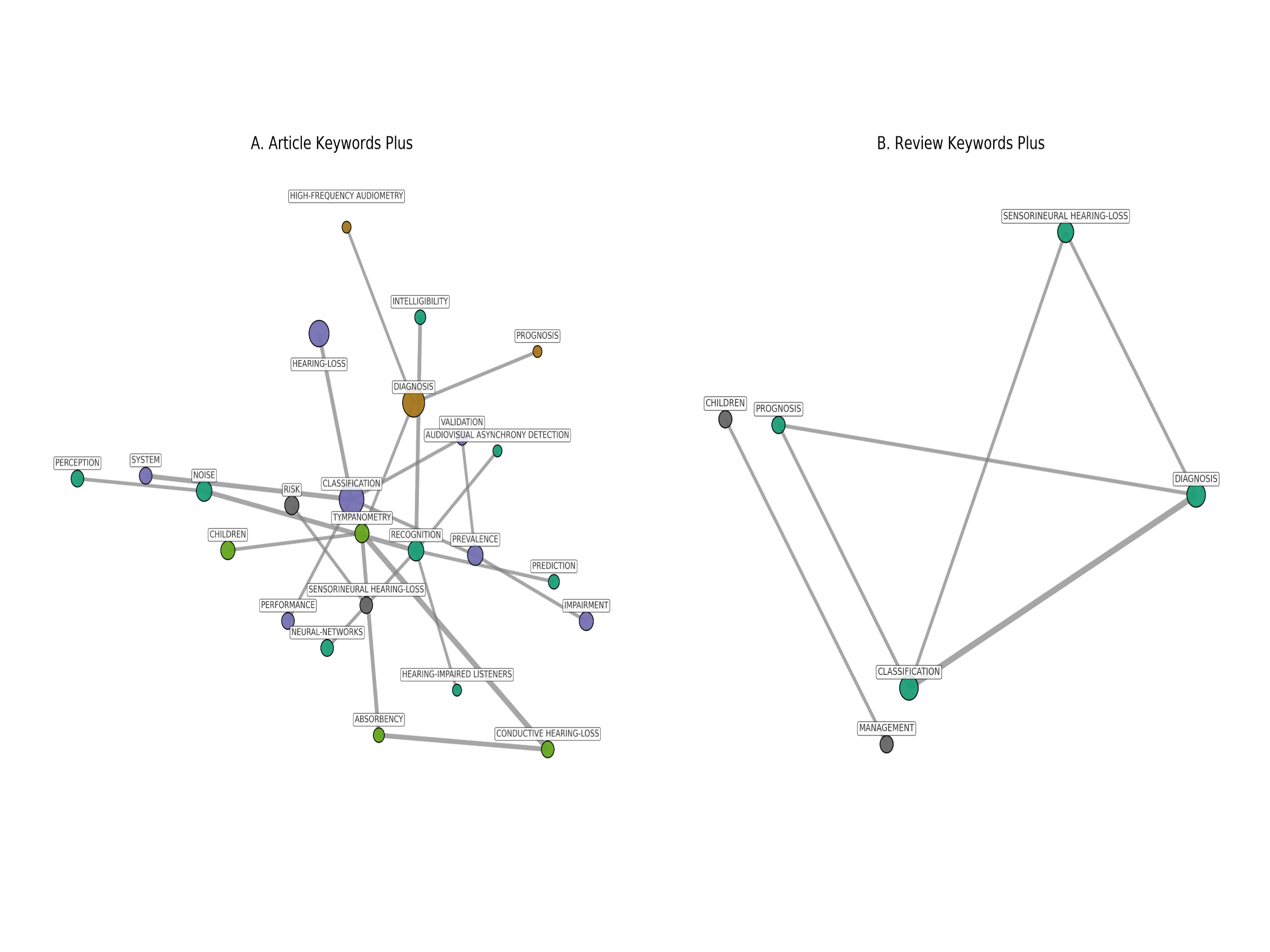

Supplement: Supplementary file 1 [file audiolres-16-00029-s001.zip › Figure S9.tiff]
